# Leukemia/lymphoma development in IL-15-deficient TCR-transgenic mice

**DOI:** 10.1186/2051-1426-3-S2-P67

**Published:** 2015-11-04

**Authors:** Sigrid Dubois, Juergen Mueller, Lionel Feigenbaum, Thomas A Waldmann

**Affiliations:** 1 Lymphoid Malignancies, Center for Cancer Research, National Cancer Institute, National Institutes of Health, Bethesda, MD, USA; 2 Center for Cancer Research, National Cancer Institute, National Institutes of Health, Bethesda, MD, USA; 3 National Cancer Institute, National Institutes of Health, Bethesda, MD, USA

## 

Sporadic mouse tumor models are valuable tools to understand disease development and treatment efficacies. Here we show that a transgenic T cell receptor expression predisposes mice to leukemia/lymphoma development. Leukemia/lymphoma precursors expressed low amounts of MHC class I and were deleted by NK cells in immunocompetent mice resulting in increased tumor frequency under NK cell absence. Leukemias/lymphomas were clonal and regrow after transfers into NK cell-deficient hosts. Phenotypically, most leukemias/lymphomas were positive for CD3 and CD8, and all expressed high levels of the co-stimulatory molecules PD1, ICOS and CD28 as well as of CD30 resembling the phenotype of immature CD8 single-positive thymocytes. Half of the leukemias/lymphomas harbored Notch1 mutations. Dendritic cells appear to have an auxiliary role in leukemia/lymphomagenesis since their deletion caused decreased tumor growth. The study of leukemias/lymphomas in IL-15-deficient TCR-transgenic mice may help to further understand T cell lymphomagenesis, the role of lymphoma environment, and may be useful to design and test treatments.

